# Genome-scale embryonic developmental profile of gene expression in the common house spider *Parasteatoda tepidariorum*

**DOI:** 10.1016/j.dib.2018.05.106

**Published:** 2018-05-24

**Authors:** Sawa Iwasaki-Yokozawa, Yasuko Akiyama-Oda, Hiroki Oda

**Affiliations:** a Laboratory of Evolutionary Cell and Developmental Biology, JT Biohistory Research Hall, 1-1 Murasaki-cho, Takatsuki, Osaka 569-1125, Japan; b Microbiology and Infection Control, Osaka Medical College, Takatsuki, Osaka, Japan; c Laboratory of Biohistory, Department of Biological Sciences, Graduate School of Science, Osaka University, Japan

## Abstract

We performed RNA sequencing (RNA-Seq) at ten successive developmental stages in embryos of the common house spider *Parasteatoda tepidariorum*. Two independent datasets from two pairs of parents represent the normalized coverage of mapped RNA-Seq reads along scaffolds of the *P. tepidariorum* genome assembly. Transcript abundance was calculated against existing AUGUSTUS gene models. The datasets have been deposited in the Gene Expression Omnibus (GEO) Database at the National Center for Biotechnology Information (NCBI) under the accession number GSE112712.

**Specifications Table**

**Table:** 

Subject area	Biology
More specific subject area	Developmental Biology
Type of data	wiggle format, tab-delimited text
How data was acquired	RNA-Seq by Illumina MiSeq
Data format	Processed
Experimental factors	Successive developmental stages, two replicates
Experimental features	mRNA from whole embryos at each stage was sequenced and the sequence reads were processed.
Data source location	Osaka, Japan
Data accessibility	The data have been deposited in the Gene Expression Omnibus database at NCBI under the accession number GSE112712.
	https://www.ncbi.nlm.nih.gov/geo/query/acc.cgi?acc=GSE112712

**Value of the data**

• The datasets are useful for identifying and characterizing transcripts from the regions of interest in the *P. tepidariorum* genome.
• The datasets are useful for examining developmental changes in expression of a gene of interest.
• The datasets may be used to characterize genes based on their expression levels and profiles.
• The datasets can be visualized in a genome browser where the *P. tepidariorum* genome assembly is embedded.

## 1 Data

The genome of *Parasteatoda tepidariorum* has been sequenced and annotated using gene models [1]. Embryonic development of *P. tepidariorum* is divided into more than 10 stages [2], [3], [4]. The datasets described here represent the normalized coverage of mapped RNA sequencing (RNA-Seq) reads along scaffolds of the *P. tepidariorum* genome assembly for ten successive developmental stages (stage (st) 1, st2, st3, st4, st5 early, st5 late, st6, st7, st8, and st10). The normalized abundance of the transcripts was calculated against the annotated gene models and is shown in a data table, together with the features of the gene models.

## 2 Experimental design, materials and methods

Two developmental series of mRNA were independently obtained from embryos produced by two pairs of parents and analyzed by RNA-Seq. Embryos were incubated at 25 °C until they reached the appropriate stages. Poly(A) mRNA was extracted using the Dynabeads mRNA DIRECT Kit (Ambion) from 10–100 embryos of each developmental stage. mRNA was fragmented using the NEBNext RNase III RNA Fragmentation Module (New England BioLabs). Sequencing libraries were constructed from the fragmented RNA using the NEBNext Ultra Directional RNA Library Prep Kit for Illumina (New England BioLabs) and NEBNext Multiplex Oligos for Illumina (Index Primers Set 1, New England BioLabs). The libraries were sequenced by Illumina MiSeq. Sequence reads were trimmed to remove the adaptor and primer sequences and low-quality sequences, using the CLC Genomics Workbench 7.0.3 (Qiagen). The parameter settings for the quality trimming were as follows: trim using quality scores, limit=0.05; trim ambiguous nucleotides, maximum number of ambiguities=2; and filter on length, discard reads below length=30. Trimmed reads were then aligned to Ptep_1.0 genome assembly (Ptep_1.0, GCA_000365465.1) using the BLAT algorithm version 34 in the DDBJ Read Annotation Pipeline (https://p.ddbj.nig.ac.jp/pipeline/) with default settings. The output alignments were filtered based on quality, coverage, and uniqueness using a PERL script filterPSL.pl available from the AUGUSTUS 3.0.1 scripts folder (http://bioinf.uni-greifswald.de/augustus/). The parameter settings were as follows: minimum coverage, 60%; minimum percent identity, 90%; uniqueness threshold, 0.96. The filtered alignments were converted into SAM files with a custom PERL script. Using the SAM files, the abundance of the mRNAs fitting AUGUSTUS gene models [1] was calculated with htseq-count v. 0.6.1p1 in -s reverse, -m union settings and was normalized in reads per kilobase of exon per million reads (RPKM). The alignments were converted into wig files using the aln2wig script available from the AUGUSTUS 3.0.1 scripts folder. Scores in the wig files represent the normalized read coverage (per 10 million reads).
